# The Interim Report of the Fabian Society
*Previous articles appeared in The Hospital of March 28 and April 4.


**Published:** 1914-04-11

**Authors:** 


### NATIONAL INSURANCE ACT DEVELOPMENTS.

### The Interim Report of the Fabian Society* (*concluded*).

In view of the importance attaching to the report of the Committee of Inquiry appointed by the Research Department of the Fabian Society, which was published as a special supplement to the *New Statesman* on March 14, we make no apology for again referring to it. We have already dealt with the sections of the report in which the administration of medical and sanatorium benefits were considered, and we now turn to the sections relating to sickness and maternity benefits.

It is noteworthy that the first paragraph of the section dealing with the administration of sickness benefit contains a tribute to the approved societies. After acknowledging the many difficulties with which the societies have had to contend, the committee record that benefits are being administered, on the whole, with justice, efficiency, and success.

In spite of this general appreciation, however, the report declares that the number of complaints by insured persons and others is very large. It is recorded that case after case has been reported in which a *bona-fide* claim has been rejected for some reason which seems to be unwarranted by either law or justice, even after due allowance has been made for the necessity of frustrating the efforts of the malingerer.

It will, of course, be obvious that with 23,500 societies administering the benefits of the Acts, differing one from another in their rules, in their management, and in the strictness of supervision of their claims, there must be differences in the results of their administration. It also seems to be clear that a certain proportion of the societies alarmed by the real or fancied excess of sickness claims have used every effort to keep down their benefit payments, and in their anxiety to do this have insisted upon the fulfilment of technical requirements that other societies have ignored. This fact is clearly evidenced in the report, and the committee apply to it the picturesque title of "nibbling" at the benefits. There are, however, allegations in the report regarding the non-admittance of claims which are far more serious, amounting in some instances to charges of direct contravention of the provisions of the Acts, and we would, therefore, draw attention to the nature of these charges.

### Claims for Benefit by Married Women.

The greater number of complaints brought to the notice of the committee were concerned with the refusal of sickness benefit to insured married women claiming for disablement caused by pregnancy. The basis of such refusal apparently lies in the fact that women's friendly societies existing before the Act of 1911 came into operation specifically excluded pregnancy as a ground for claim, and it has accordingly been taken for granted by many officials accustomed to friendly society work that pregnancy also gives no ground for claim under the Insurance Acts. It is difficult to see how such a refusal of benefit can be in any way justified, since the Act of 1911 definitely states, without qualification, that sickness benefit is payable in cases of incapacity for work, whether such incapacity is caused by specific disease or by *bodily or mental disablement.* In face of this, approved society officials are to blame if they have acted according to custom, as custom has now been overruled by a statutory provision. It is unfortunate that the Commissioners have not put matters right by issuing an official regulation on the subject, but the absence of such regulation does not relieve the responsibility of societies. It may be mentioned in passing that the Commissioners have in some cases, in replying to inquiries, definitely laid it down "that sickness benefit is payable in cases of simple pregnancy as in those of any other disablement, *provided there is incapacity to work*, exactly as in cases of specific disease." Any attempt, therefore, to withhold benefit in such circumstances should be protested against as being contrary to the provisions of the Acts, and any society which retains a rule excluding cases of pregnancy from eligibility for sickness benefit "ought to be required, so far as regards the State benefit, to expunge such rule as *ultra vires*."

### What is Meant by "Incapable of Work"?

Unfortunately, the difficulty regarding pregnancy cases is not altogether removed, even when it is understood that disablement arising from pregnancy is to be treated in the same way as disablement resulting from specific disease. A further difficulty has arisen over the interpretation of the phrase "incapable of work." This difficulty is, of course, not confined to cases of pregnancy, but has a much more far-reaching effect, and has accordingly received very full consideration in the report. The trouble arises from the fact that the phrase "incapable of work" may be construed as meaning either "incapable of following his ordinary employment" or "incapable of any work whatsoever." It is safe to say that the first interpretation is held by almost every one of the millions of insured persons to be the meaning of the phrase, but unfortunately the second appears to be the official definition. Societies wishing to safeguard their funds have naturally favoured the official definition, and in some cases this adherence to official interpretation has led to a refusal of benefit which is obviously contrary to the spirit of the Acts. It will be perfectly clear that a person who is only to receive benefit when "incapable of any work whatsoever" is very unlikely to receive any benefit at all. The report truly points out in a most trenchant criticism of this definition that it practically amounts to providing benefit only when the insured person is unconscious, as except when he

* Previous articles appeared in The Hospital of March 28 and April 4.

is in that condition it could in most cases be held that he was able to perform some trifling work, no matter how ill he might be. The chief instances of hardship have occurred in connection with claims for benefit made by married women. Certain approved societies have repeatedly refused to pay a claim because the woman has, when ill, performed some light household duties. To object to the performance of heavy household work is reasonable enough, but it is carrying matters to an extreme when objection is raised to the performance of light work.

Whatever the official definition of the phrase, it is perfectly clear that it is inequitable to consider it as meaning literally "incapable of any work whatsoever," and, apart from equity, it would not be to the advantage of approved societies, in the long run, to adhere to such a definition. A man may be quite *capable* of undertaking work of some description yet be ill-advised to do so, as its performance might retard or even prevent his recovery. Such retardation or prevention would obviously operate against the interest of the societies, and in many cases the result would be the payment of sickness benefit for a period far in excess of that which would ordinarily mark the duration of the illness.

There is, on the other hand, a danger in following too closely the wider definition. If a person becomes so disabled as not to have any hope of resuming his normal occupation, it would be unfair to his society for him to receive sickness and disablement benefit until his death or attainment of the age of seventy, unless he is actually incapable of performing any work without further prejudicing his health. It would be his duty in such circumstances to change his employment and find some work within his modified powers.

Considering the matter in all its aspects, it would seem, as pointed out by the committee, that "it is necessary in practice to make a distinction between merely temporary disablement, during which the patient may reasonably be supposed to be going one day to be able to resume his ordinary employment, and disablement which must be recognised as rendering it necessary for him to change his work, and that as a general rule all disablement must at the outset be presumed to be temporary only." Sickness benefit should be payable in the case of temporary disablement if it were clear that any attempt on the part of the insured person to follow his ordinary employment would be prejudicial to his recovery.

### The Administration of Maternity Benefit.

With regard to maternity benefit, the report confirms the generally accepted opinion that this benefit, especially since the coming into force of the Amendment Act of 1913, is the most popular and the least unsatisfactory of all the benefits of the Acts.

Two drawbacks to which attention is directed are (1) that almost invariably the wives of persons employed in "excepted" employments are altogether excluded from any maternity allowance, and (2) that the wives of deposit contributors stand very little chance of ever securing maternity benefit in full. Both these grievances are undoubtedly real, but can perhaps not be regarded as other than more or less minor defects of the National Insurance scheme.

Another and a much more serious point is, however, brought to light by a statement in the report that in many parts of the country the doctors (and in some cases the midwives) have seized the opportunity of the payment of the maternity benefit very greatly to increase their charges for midwifery. This is undoubtedly a serious matter, and if the practice is carried on to any extent the value of the maternity benefit to insured persons is being very largely lost. We can only express the hope that the allegations are exaggerated, and if they are not, the matter should be made the subject of most careful inquiry.

### Conclusions of the Committee.

Our discussion of this comprehensive and instructive report would be incomplete without some reference to the general conclusions drawn by the committee from the facts brought before them. The proposals outlined at the end of the report for the improvement of the National Insurance scheme are in the main of a sweeping character, and are naturally coloured with the political and social views of the promoters of the investigation. To do them justice, nevertheless, the committee put forward very strong arguments in justification of each of their suggestions, and their conclusions are, therefore, not to be lightly dismissed.

We do not pretend to agree with all the suggestions that are made, or even with the majority of them in their entirety, but they are worthy of careful attention as the considered judgment of those who have given the matter close study. Without actually putting the idea forward as a practical suggestion, the committee indicate that in their opinion the great majority of the existing defects would be remedied if all the approved societies were abolished and the entire scheme controlled by the State. Even the suggestions that are put forward as being immediately practicable are mainly based on this same idea of State intervention. It is suggested, for example, that maternity benefit, sickness benefit in cases of pregnancy, the treatment of tuberculosis, and the treatment of venereal diseases should be removed from Insurance and placed under the control of health authorities, and that, moreover, these benefits should be available for insured and uninsured alike. It is further suggested that salaried medical referees should be appointed and paid by the Insurance Commissioners, so that a valuable and unbiassed "second opinion" should be available when required and the interests of panel doctors, approved societies, and insured persons alike served. Some valuable suggestions are also made with regard to improvements in certification, and the report concludes by indicating the necessity for administrative and constitutional reforms.

